# Free air after laparoscopic hysterectomy; from sigmoid perforation to upper airway compromise: a case report

**DOI:** 10.1186/s13256-020-02627-y

**Published:** 2021-01-25

**Authors:** Aikaterini Melemeni, Aliki Tympa Grigoriadou, Athanasia Tsaroucha

**Affiliations:** 1st Department of Anesthesiology, Aretaieion University Hospital, National and Kapodistrian University of Athens, 76 Vasilisis Sofias Avenue, 11528 Athens, Greece

**Keywords:** Laparoscopic hysterectomy, Neck edema, Case report, Ectopic air

## Abstract

**Background:**

Free air after laparoscopic hysterectomy is a common finding; in rare cases, free air represents gastrointestinal perforation, requiring emergency laparotomy. Ectopic air localizations after pneumoperitoneum have been reported in various laparoscopic surgical techniques. Delayed diagnosis of visceral perforation is associated with high mortality rates.

**Case presentation:**

We present a white Caucasian female in which dysphonia due to air entrapment in the cervical area, pneumomediastinum and pneumothorax, occured afterlaparoscopic hysterectomy.

**Conclusions:**

Upon mobilization of the patient, air from sigmoid perforation moved cephalad. Through the same path, pneumoperitoneum, causes subcutaneous emphysema in the neck and face, pneumomediastinum and pneumothorax.

## Background

Minimally invasive gynecologic surgery has often been shown to offer better clinical outcomes. The percentage of laparoscopic hysterectomies has therefore greatly increased worldwide, with reported better patient experience, early return to normal activities and shorter hospital stays.

Ectopic air localizations after pneumoperitoneum have been reported in various laparoscopic surgical techniques, including inguinal hernia repair, Nissen fundoplication and sigmoid resection.

We present a case in which, a patient suffered dysphonia, pneumomediastinum and pneumothorax, due to air entrapment after laparoscopic hysterectomy.

## Case presentation

A 45-year old white Caucasian female, with American Society of Anesthesiology (ASA) physical status 1 (57 kg, 169 cm), presented for elective laparoscopic hysterectomy. After premedication with midazolam 1 mg intravenously, anesthesia was induced with fentanyl 100 μg, propofol 180 mg and rocuronium 60 mg. Tracheal intubation was easily performed (Cormack and Lehane grade 1). Anesthesia was maintained with sevoflurane in O_2_/Air (50%:50%) and intermittent doses of fentanyl 50 μg. Muscle relaxation was achieved with 10 mg of increments of rocuronium. Standard monitoring included electrocardiography, non-invasive blood pressure, oxygen saturation (SpO_2_) and end-tidal carbon dioxide (ETCO_2_). Multigas analysis was used throughout the operation.

The patient was hemodynamically stable throughout the operation. The abdomen was insufflated blindly with carbon dioxide at a pressure of 15 mmHg. SpO_2_ was 98–99% and ETCO_2_ was maintained at less than 40 mmHg with peak inspiratory pressure between 18 and 27 cm H_2_O. Tidal volume was set at 570 mL and 14 breaths per minute respectively. The surgical procedure was prolonged, lasting 240 minutes. The patient was extubated without difficulty.

The patient was not mobilized on the first postoperative day due to pain; pain was alleviated with paracetamol 1000 mg and diclofenac 75 mg and the patient was able to walk on the next morning. Three hours after mobilization, the patient became dyspnoic; dyspnoea was worse in the supine position and by the next 15 minutes, she could maintain an SpO_2_ of 93%, only by Venturi mask of 30% FiO_2_ at an oxygen flow rate of 12 litres per minute and only in the sitting position. The patient readily developed dysphonia, neck and face edema that were initially treated as anaphylactic reaction. No improvement was seen, and the patient was transferred to the Intensive Care Unit (ICU). Crepitus could be felt in the neck, thorax and lower limb area. Chest X-ray was not diagnostic. The patient rapidly developed cardiovascular and respiratory impairment and was transferred to the operation theatre under inotropic support for emergency laparotomy. Intubation was achieved with an endotracheal tube of 5.5 mm internal diameter. Mechanical ventilation was copious with the patient deteriorating; suspected tension pneumothorax was treated with immediate decompression of the pleural space with needle thoracocentesis. Faecal odour was noticed on thoracocentesis and surgical exploration revealed sigmoid perforation which had taken place during the laparoscopic hysterectomy. The ruptured sigmoid was closed and a diagnosis of septic shock with subcutaneous emphysema caused by colon perforation was retained. The patient was transferred to the ICU, where she developed high fever and mediastinitis, 7 hours after surgery. Aggressive intravenous antibiotic therapy was started with clinical improvement during the next 3 days. The patient was extubated on the second postoperative day and was dismissed from hospital 10 days later, after the resolution of mediastinitis and infectious pneumonia. The patient suffered recurrent mild pneumonias over the next period and was completely symptom-free from the respiratory tract, two months after surgery.

## Discussion and conclusions

The complications of pneumoperitoneum have been extensively studied with variable rates depending on the type of procedure and the experience of the surgeon. Hypercapnia, subcutaneous emphysema, pneumothorax and pneumomediastinum are among the most common complications of laparoscopic surgery. Pneumoperitoneum accounts for hemodynamic instability, altered pulmonary physiology, elevated intracranial and intra-ocular pressures and thromboembolic complications. Patient-related risk factors include abdominal adhesions due to surgery, obesity, anatomic variations, inflammatory syndromes or diaphragmatic hernia. Among laparoscopic operations, fundoplication, vagotomy, urologic procedures and adrenalectomy, are considered high risk procedures. Laparoscopic hysterectomy has been reported to be a safe and complication-free surgical treatment. Table 1 summarizes the cases of ectopic air localizations and their complications after laparoscopic hysterectomy.

**Table 1.** Summary of cases of ectopic air localizations and their complications after laparoscopic hysterectomy

Author	Complication	Time of diagnosis	Symptom	Diagnostic tool	Evolution
Patti *et al.* [2]	Sc emphysema, pneumomediastinum, pneumothorax	2nd postoperative day	Facial, neck, upper and lower extremity, torso crepitus	CT	Spontaneous resolution of symptoms within a month
Saeed *et al.* [3]	Sc emphysema, pneumomediastinum	Intraoperatively	CO_2_ overload	CT	Intubation and mechanical ventilation for the first postoperative day
Vetter *et al.* [4]	Lower extremity subcutaneous edema	1st postoperative day	Tender crepitus, bruising of the right ankle	Rö	Spontaneous resolution
Fedun *et al.* [5]	Sc emphysema, bilateral tension pneumothorax, pneumomediastinum, pneumoperitoneum	After extubation	Dyspnoea, abdominal distention	CT	Thoracic drainage, mechanical ventilation for 2 days postoperatively

Sc: subcutaneous, CT: computed tomography, Ro: Radiography

In the present case, the patient has not been operated in the past and had a BMI of 20. On extubation, the patient had no subcutaneous emphysema or respiratory compromise. Kuhn *et al*. [1] recently described two cases of extraperitoneal diverticulum perforation with similar clinical presentation. Dysphonia would prompt for allergic reaction whereas subcutaneous emphysema, recognized later in the course of the patient would prompt for the well-known complications of the laparoscopic surgery. The small amount of subdiaphragmatic air at the chest X-ray was attributed to the pneumoperitoneum of the laparoscopic hysterectomy. In a rough diagnostic evaluation, the first of the patient symptoms would guide us towards laparoscopic surgery and its relatively easy treated complications. It has been stated that postlaparoscopic pneumoperitoneum resolves within three days in 81% of the patients [2, 3]; It should be underlined though that normal excretion of CO_2_ is 100–200 mL/minutes and is increased by 14–48 mL/minutes when CO_2_ is administered intraperitoneally. CO_2_ has a high solubility and thus complications like capnothorax, subcutaneous emphysema, pneumothorax and pneumomediastinum due to laparoscopy are expected to occur within the 24 hours after laparoscopic surgery.

Risk factors like prolonged duration of pneumoperitoneum and elevated insufflation pressure—which are common in laparoscopic gynecologic surgery—can erroneously guide the diagnosis towards the complications of laparoscopy absorbed CO_2_, instead of the silently progressive septic shock due to gut perforation. Furthermore, the fact that the patient was not mobilized on the first day, possibly masked the symptoms of the free air in the peritoneal cavity; air from sigmoid perforation moved cephalad upon mobilization and spread through the same path pneumoperitoneum causes subcutaneous emphysema in the neck and face, pneumomediastinum and pneumothorax.

## Data Availability

Not applicable.
